# Polysaccharides from the Cherry Peel of *Coffea arabica* L. Attenuate Obesity by Altering Lipid Metabolism and Inflammation and Regulating Gut Microbiota in Mice Fed a High-Fat Diet

**DOI:** 10.3390/foods15020312

**Published:** 2026-01-15

**Authors:** Guiqin Hu, Yinghong Gu, Wenyang Zhang, Xiaobin He, Xingzhong Wu, Yufei Jiang, Hong Li, Yu Cao

**Affiliations:** 1 College of Food Science and Technology, Yunnan Agricultural University, Kunming 650201, China; huguiqin1001@163.com (G.H.); m15108764168@163.com (Y.G.); 13638771610@163.com (W.Z.); bin_20311@163.com (X.H.); wuxingzhong0210@163.com (X.W.); jlckca793rz1@163.com (Y.J.); 2 Yunnan College of Coffee Modern Industry, Yunnan Agricultural University, Kunming 650201, China; 3 Yunnan Baoshan Coffee Technology Institute, Yunnan Agricultural University, Baoshan 678000, China

**Keywords:** polysaccharides, coffee cherry peel, obesity, gut microbiota, anti-inflammatory, antioxidant, short chain fatty acids

## Abstract

Long-term excessive fat intake can easily induce metabolic diseases such as fatty liver and hyperlipidemia. As a natural active ingredient, polysaccharides exhibit notable lipid-lowering effects and can serve as effective lipid regulators. Nevertheless, the lipid-lowering effect of Arabica coffee cherry peel polysaccharides (CCPPs) and the underlying regulatory mechanism remain poorly understood. This study isolated polysaccharides from coffee cherry peel, and their functional properties and the lipid-lowering effects and mechanisms on hyperlipidemic mice. In high-fat diet-fed (HFD-fed) mice, CCPP administration had significant regulatory effects on various metabolic parameters. In laboratory mice where hyperlipidemia is induced by a high-fat diet, CCPP administration improved serum lipid levels and demonstrated anti-inflammatory and antioxidant effects. These benefits were achieved by reducing pro-inflammatory cytokine expression, enhancing antioxidant enzyme activity, and lowering overall oxidative stress. Additionally, it effectively decreased fat area in liver tissues and adipocytes. Specifically, compared with the control group, after high-dose CCPP intervention, the adipocyte area of mice on a high-fat diet was significantly reduced by 41.3%. Notably, CCPP intervention resulted in a shift in the gut microbiota composition. At the phylum level, the model group showed a significant increase in Bacillota and a concomitant reduction in Bacteroidetes in comparison with the control group. Compared with the model group, CCPP intervention, especially in the CCPP-H group, resulted in an increase in the proportion of Bacteroidetes and a decrease in Bacillota. At the genus level, CCPP modulated the abundances of key bacterial genera; for instance, the relative abundance of *Lachnospiraceae_NK4A136_group* increased from 2.64% in the model group to 11.9% in CCPP-H group, while *Faecalibaculum* decreased from 62.69% to 41.27% in CCPP-L group and 25.29% in CCPP-H group. These shifts suggest that CCPP has a reparative effect on the gut microbial composition, potentially contributing to the promotion of gut health. Taken together, these factors highlight the promise of CCPP as a functional food ingredient for dietary interventions to ameliorate obesity and hyperlipidemia.

## 1. Introduction

The global obesity epidemic continues to worsen, with data indicating that the prevalence of obesity has increased eight- to ninefold over recent decades [1]. To some extent, obesity can be regarded as a disease, with its core mechanism rooted in metabolic disorders caused by long-term energy imbalance (energy intake > energy expenditure). Obesity often elevates the risk of conditions like type 2 diabetes and cardiovascular ailments and non-alcoholic fatty liver disease (NAFLD), ultimately posing a serious threat to the healthy lifespan of populations [2,3,4]. NAFLD is not a simple disease process. It is defined as an acquired liver injury characterized by excessive hepatic fat accumulation, which is attributed to metabolic stress rather than to alcohol or other known hepatotoxic factors. It is closely associated with metabolic syndrome, manifesting as components of metabolic syndrome such as dyslipidemia, hypertension, hyperglycemia, and obesity [5,6]. In its early stages, NAFLD primarily presents as steatosis. Sustained oxidative stress and inflammation may exacerbate liver injury, advancing to non-alcoholic steatohepatitis (NASH) with increased risks of cardiovascular disease and cancer [7].

**Figure 1:**
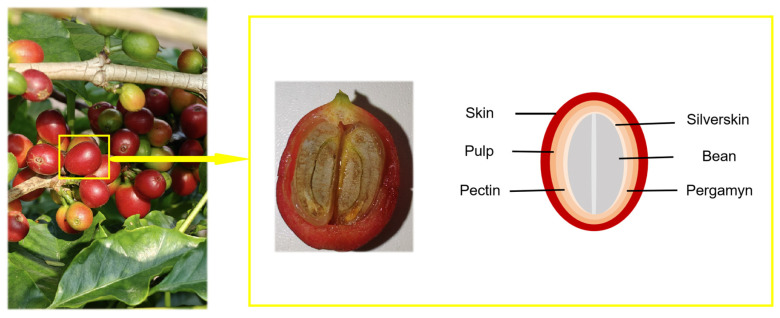
Cross-sectional diagram of coffee cherry fruit.

In recent years, with the rise in living standards and shifts in dietary patterns, the prevalence of obesity has continued to rise, and weight management has garnered increasing attention. Currently available weight-loss medications on the market primarily include statins, orlistat [8], liraglutide and semaglutide [9], naltrexone/bupropion [10], and phentermine/topiramate [11], among others. However, their clinical application is frequently limited by gastrointestinal side effects such as steatorrhea and nausea, potential long-term safety concerns, and high costs, which collectively restrict patient adherence and accessibility. These constraints highlight the urgent need to explore safer, more tolerable, and cost-effective alternative strategies. In this context, plant-derived polysaccharides have garnered considerable interest due to their multifunctional bioactivity, favorable safety profile, and potential to modulate core pathological processes in obesity such as gut microbiota dysbiosis and chronic inflammation. Accumulated evidence from multiple studies indicates that natural plant polysaccharides exert beneficial effects on lipid metabolism [12,13,14]. As a natural active substance, plant polysaccharides demonstrate favorable safety and efficacy. A substantial amount of by-products are generated during coffee production, and roughly 50% of the material, largely comprising peels, is routinely discarded. The valorization of coffee peel containing skin, pulp, and pectin presents an opportunity to enhance the economic returns of the coffee industry while mitigating environmental pollution and resource wastage. As a rich source of polysaccharides, coffee peel has great application prospects. While existing research has noted its antioxidant capacity and emulsifying properties, the specific composition, functional activities such as the hypolipidemic activity, and mechanistic details of coffee peel polysaccharides are still not fully understood. A cross-sectional diagram of coffee cherry fruit is illustrated in Figure 1.

In this study, polysaccharides were extracted and purified from the peel of coffee cherries and named CCPP. The effects of CCPP in alleviating obesity were evaluated in a mouse model of high-fat diet-induced obesity. We also examined the hypothesized mechanisms by which CCPP reduces obesity and hyperlipidemia by controlling hepatic lipid metabolism, inflammatory mediators, gut microbiota, and metabolic products. These results will offer fresh proof for the creation and use of CCPP resources and lay the foundation for future investigations on the bioactivity mechanisms of polysaccharide components.

## 2. Materials and Methods

### 2.1. Experimental Materials and Reagents

Fresh coffee cherry peel from Yunnan *Coffea arabica* was obtained from Lujiangba in Longyang District, Baoshan City, Yunnan Province, China. The TNF-α kit was bought from Beijing Solarbio Technology Co., Ltd. (Beijing, China). Additional kits were acquired from Nanjing Jiancheng Biotechnology Research Institute. All other biological reagents and chemical reagents were of analytical grade and were purchased from Shanghai Yuanye Bio-Technology Co., Ltd. (Shanghai, China). We purchased male C57BL/6 mice from Sibefu (Beijing) Biotechnology Co., Ltd. (Beijing, China). A high-fat diet was obtained from Jiangsu Collaborative Pharmaceutical Biotechnology Engineering Co., Ltd. (Nanjing, China). The animal study received ethical clearance from Yunnan Agricultural University’s Animal Ethics Committee (approval number APYNAUSQ 202502009).

### 2.2. Extraction, Separation, and Purification of CCPP

To derive crude polysaccharides from fresh coffee cherry peel, 60 g of the coffee cherry peel was soaked in distilled water at a solid-to-liquid ratio of 1:15 (*w*/*v*) for 30 min. The mixture was then homogenized using a juicer and subjected to ultrasonic treatment for 1 h. The temperature during ultrasonic extraction was kept at 65 °C. The ultrasonicated mixture was clarified by filtration, and the filtrate was retained. The filtrate was concentrated by rotary evaporation and then mixed with 80% ethanol, followed by overnight standing at 4 °C to allow precipitation [15,16,17,18,19]. Next, the blend was spun in a centrifuge at 4000 revolutions per minute for a solid 10 min, allowing the sediment to settle at the bottom. The collected precipitate then underwent freeze-drying to extract raw polysaccharides straight from fresh coffee cherry husks.

To remove proteins from the crude polysaccharides, the Sevag method was employed for deproteinization [20,21,22]. The CCPP was dissolved in distilled water and subjected to DEAE-52 cellulose column chromatography (4 × 65 cm) for elution. A concentration gradient of NaCl solutions (0.1, 0.2, 0.3, 0.4, 0.5, and 0.6 M), along with distilled water, served as the eluents, with elution carried out at 1 mL/min. The polysaccharide content in the eluate was quantified utilizing the phenol–sulfuric acid method, yielding neutral polysaccharides (distilled water, CCPP-0) and acidic polysaccharides (0.1 M NaCl, CCPP-1). The CCPP-0 was further purified using a Sephadex G-200 gel filtration column (2 × 80 cm) and elution was performed at a rate of 0.5 mL/min, with fractions of 5 mL subsequently collected. Total sugar was quantified for all collected fractions via the phenol–sulfuric acid method, based on spectrophotometric absorbance readings at 490 nm [23]. Appropriate eluates were collected based on the peaks in the absorbance elution curve, dialyzed using a dialysis bag with a molecular weight cutoff of 3500 Da, and the dialysate was concentrated and followed by lyophilization to obtain the final polysaccharide.

### 2.3. Determination of Molecular Weight and Monosaccharides Analysis

The molecular weight of coffee peel polysaccharides was determined by referring to the methods described in references [24,25,26,27]. For analysis, 1 mg/mL polysaccharide solution was prepared by dissolution in a 0.1 M aqueous NaNO_3_ solution containing 0.02% (*w*/*w*) NaN_3_. The initial solution underwent filtration using a 0.45 μm membrane before being introduced into the analytical instrument. To nail down the monosaccharide profile of coffee peel polysaccharides, we put the sample through HPLC analysis, sticking to tried-and-true methodologies [28,29,30,31]. The ion chromatography system used to analyze monosaccharide components is Thermo ICS 5000+ of Thermo Fisher Scientific (Waltham, MA, USA), which has an electrochemical detector. Dionex™ CarboPac™ PA20 column (150 × 3.0 mm, 10 μm) was used for separation. Each injection volume is 5 μL. The mobile phase includes (a) water, (b) 0.1 M sodium hydroxide solution, and (c) mixed solution of 0.1 m sodium hydroxide and 0.2 M sodium acetate. The flow rate was 0.5 mL/min and the column temperature remained constant at 30 °C. The gradient elution procedure is as follows: 0 min (A:B:C = 95:5:0, volume ratio), 26 min (85:5:10), 42 min (85:5:10), 42.1 min (60:0:40), 52 min (60:40:0), 52.1 min (95:5:0), and 60 min (95:5:0). Individual stock standard solutions (10 mg/mL) were first prepared by accurately weighing the required monosaccharide standards, comprising fucose, rhamnose, arabinose, galactose, glucose, xylose, mannose, fructose, ribose, galacturonic acid, glucuronic acid, mannuronic acid, and guluronic acid. Subsequently, these standard solutions were mixed to create mixed standard solutions with varying concentrations (up to 60 μg/mL). The sample preparation steps were as follows: a precise amount of the polysaccharide sample was accurately weighed out for subsequent analysis. An appropriate amount of polysaccharide sample was taken, and we added 1 mL of 2 M trifluoroacetic acid (TFA) mixture. The mixture was heated at 121 °C for 2 h. Afterward, the solution was dried under nitrogen gas, washed with 99.99% methanol, and dried again. This methanol washing step was repeated 2–3 times. Finally, the residue was dissolved in sterile water and prepared for analysis. We used Thermo ICS 5000+ ion chromatograph for analysis, which was equipped with an electrochemical detector and Dionex™ CarboPac™ PA20 column. Next, we added 5 μL of sample to the system at each injection. We used three solvents, A (water), B (0.1 M sodium hydroxide), and C (0.1 M sodium hydroxide plus 0.2 M sodium acetate mixture), and the flow rate was 0.5 mL/min. The temperature of the chromatographic column was kept at 30 °C and a specific elution gradient was used.

### 2.4. Animal Experiment Design

**Figure 2:**
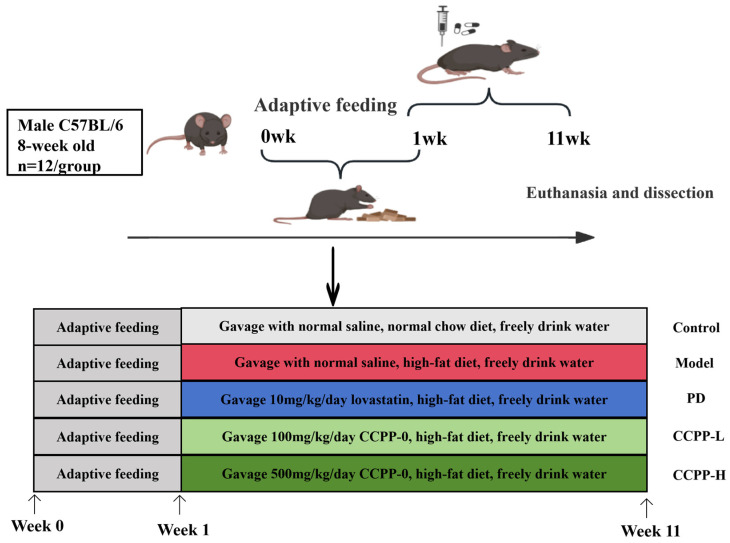
Mouse experiment design diagram. Grayish-white represents the normal control group, red represents the high-fat diet group (the model group), blue represents the positive drug group (high-fat diet, gavage administration of 10 mg/kg/day lovastatin), light green represents the low-dose group of coffee cherry peel polysaccharides (high-fat diet, gavage administration of 100 mg/kg/day CCPP-0), and dark green represents the high-dose group of coffee cherry peel polysaccharides (high-fat diet, gavage administration 500 mg/kg/day CCPP-0).

We divided 60 male C57BL/6 mice into five groups: one group ate normal food (Control) and the other group ate high-fat food (Model). The other groups were the positive drug lovastatin group (PD), the high-fat diet + polysaccharide low-dose group (CCPP-L), and the high-fat diet + polysaccharide high-dose group (CCPP-H). The mice were given a one-week adaptation feeding period. Next, the subjects were fed high-fat food for ten weeks so that the Model could be made, and CCPP-0 was administered throughout the course of high-fat diet feeding. The doses were 10 mg/kg/day lovastatin for the PD group, 100 mg/kg/day CCCP-0 for the CCPP-L group, and 500 mg/kg/day CCPP-0 for the CCPP-H group. The mice lived in an environment of 25 °C, with 12 h of light and 12 h of darkness every day, and food and water could eaten at will. the weight of mice was recorded every week. After being given oral drugs, the experimental animals were euthanized, and samples were collected and preserved for subsequent analysis. A schematic diagram of the whole experimental step can be seen in Figure 2.

### 2.5. Collection of Tissue Samples

Aseptic collection of fecal samples from each mouse was initiated three days before the experimental endpoint. After the experiment ended, the mice were starved all night, but they could drink water at will. After cervical dislocation, blood was collected via eyeball extraction. Multiple tissue specimens, including the spleen, colon (plus its contents), cecum contents, liver, inguinal white adipose tissue, and epididymal white adipose tissue were harvested and archived at −80 °C for future use. A portion of the adipose tissue and liver tissue was fixed in 10% paraformaldehyde for routine morphological analysis. After sitting out at room temperature for roughly 3–4 h, the blood sample was rotated in the centrifuge at a speed of 5000 rpm for 10 min, and the whole process was carried out at a low temperature of 4 °C. We carefully collected the top liquid part and put it in a freezer at −80 °C so that it could be kept fresh and used for analysis later.

### 2.6. Measurement of Serum Biochemical Index, Inflammatory Factors, and AST and ALT Enzyme Activities

According to the instructions of the corresponding kits (kits were bought from Nanjing Jiancheng Biotechnology Research Institute), the levels of serum total cholesterol (TC), triglycerides (TG), high-density lipoprotein cholesterol (HDL-C), and low-density lipoprotein cholesterol (LDL-C) were measured. We used an enzyme-linked immunosorbant assay (ELISA) to measure the concentrations of several inflammatory substances in the blood—TNF-α, IL-1β, and IL-6. All experiments were carried out using the kits purchased from Shanghai enzyme-linked biotechnology Co., Ltd. in Shanghai, China. According to the manufacturer’s instructions, we measured the serum levels of aspartate aminotransferase (AST) and alanine aminotransferase (ALT) with a special detection kit from Nanjing Jiancheng Bioengineering Institute in China. All operational steps were performed in strict accordance with the equipment manufacturer’s guidelines.

### 2.7. Measurement of Hepatic Antioxidant Indicators

We measured the protein content in liver homogenate samples via the method provided by the BCA assay kit, The BCA kit was purchased from Beijing Solabio Co., Ltd. (Beijing, China). According to the instructions in the kit from Nanjing Jiancheng Bioengineering Institute (Nanjing, China), we measured the concentrations of MDA, GSH, T-AOC, CAT, GSH-Px and SOD in the liver mixture.

### 2.8. Histopathological Analysis

Samples of white adipose tissue of liver and epididymis were first soaked in 10% formaldehyde solution and fixed. Then, we dehydrated them with higher and higher concentrations of ethanol. Then, these samples were treated with xylene to make them transparent, and finally they were embedded with paraffin. The tissues were cut into pieces. Each piece was only 4 to 5 microns thin. Then, according to the standard method of the laboratory, the paraffin was removed, and then the samples were dyed with hematoxylin and eosin (H&E). After dyeing, we dehydrated the tissue samples, fixed them on a glass slide with a glass cover, and then observed them through an optical microscope for in-depth histological examination.

### 2.9. Determination of SCFAs in Mouse Cecal Contents

A 20 mg specimen was precisely measured with 500 μL of methanol–water (4:1, *v*/*v*) extraction solution added. The mixture was ground using a cryogenic grinding apparatus for 6 min (−10 °C, 50 Hz), followed by ultrasonic treatment at low temperature for 30 min (5 °C, 40 kHz). The mixture was allowed to stand at −20 °C for 30 min, then centrifuged at 4 °C and 13,000 rcf for 15 min. We took 20 μL of the supernatant and added 20 μL of 200 mM 3-nitrophenylhydrazine hydrochloride (3-NPH·HCl) and 20 μL of 120 mM 1-ethyl-3-(3-dimethylaminopropyl) carbodiimide hydrochloride (EDC·HCl) solution (containing 6% pyridine). The derivatization reaction was then incubated at 40 °C for 30 min. Finally, we diluted the mixture to 1000 μL with 50% acetonitrile aqueous solution before instrumental analysis.

### 2.10. Microbiome Analysis

After collection, we first froze the feces of rats at below zero and then checked the samples. Then, we used a standard gene extraction kit (GHFDE100, Hangzhou Guhe Information Technology Co., Ltd., Hangzhou, China) to extract DNA from these samples. We used NanoDrop 2000c spectrophotometer (Thermo Scientific, Waltham, MA, USA) for quantitative analysis. Target amplicons were excised from 2% agarose gels and purified using the AXYGEN Gel Extraction Kit (AP-GX-250G, Axygen Biosciences, Union City, CA, USA). The purified PCR products were quantified with the Qubit dsDNA HS Assay Kit (Q32851, Life Technologies, Carlsbad, CA, USA). Sequencing libraries were prepared using the KAPA Library Quant Kit (KK4824, KAPA Biosystems, Wilmington, MA, USA) and subsequently sequenced on an Illumina NovaSeq 6000 platform (300-cycle S4 reagent cartridge). Raw sequencing reads were processed through the QIIME pipeline (v1.8.0) for quality filtering and downstream analysis. Alpha diversity was assessed using Chao, Shannon, Simpson, and Abundance-based Coverage Estimator (ACE) indices to quantify the microbial community’s richness (Chao, ACE) and evenness (Shannon, Simpson) within samples. Beta-diversity analysis, utilizing techniques such as PCoA and NMDS, was conducted to evaluate dissimilarities among bacterial communities [32,33,34,35]. The linear discriminant analysis effect size (LEfSe) method was used to analyze the abundance of various microorganisms in the 16S rRNA gene sequencing data. [36,37]. Spearman correlation analysis was employed to examine the relationships between gut microbiota and oxidative stress, inflammation, and metabolic activity, and to determine their associations with a high-fat diet.

### 2.11. Statistical Analyses

Data were analyzed using one-way ANOVA with GraphPad Prism 10 and SPSS 27.0 software. Before performing one-way ANOVA, we used the Shapiro–Wilk test to check whether the data conformed to the normal distribution. The results were expressed by mean and standard deviation. Multiple comparisons were performed using Dunnett’s test, with a *p*-value of <0.05 set as the significance threshold. Graphical representations of the data were prepared using Origin 2024, BioGDP.com, and GraphPad Prism 10.

## 3. Results

### 3.1. Isolation, Purification, and Basic Characterization of Polysaccharides from Arabica Coffee Cherry Peel

**Figure 3:**
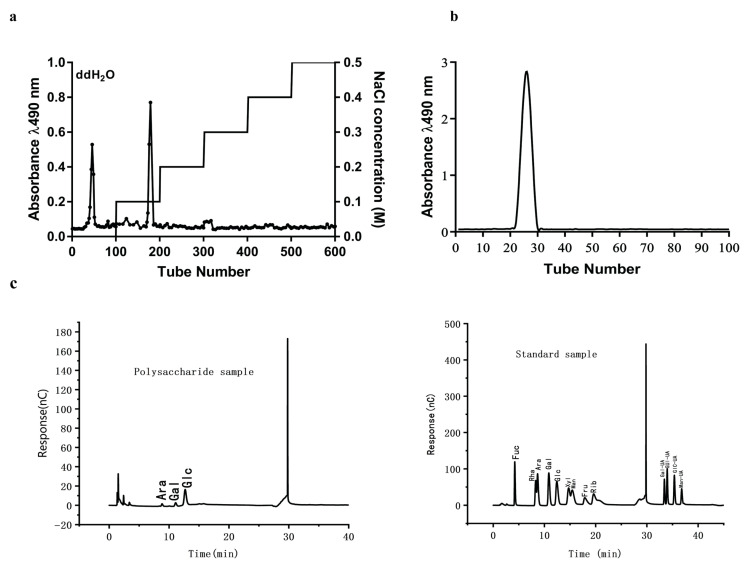
Isolation, purification and basic characterization of polysaccharides from arabica coffee cherry peel. (**a**) DEAE–cellulose elution curve. The first peak represents the component CCPP-0 eluted by ddH_2_O, and the second peak represents the component CCPP-1 eluted by 0.1 M NaCl.; (**b**) Sephadex G-200 elution curve; (**c**) monosaccharide composition of CCPP-0.

To further purify CCPP, anion-exchange chromatography was performed using a DEAE-52 cellulose column. Based on the differential binding affinities of polysaccharides, neutral sugars (with high water solubility) were eluted first, while acidic sugars (with low water solubility) required higher ionic strength for elution. As shown in Figure 3a, gradient elution was carried out sequentially with deionized water and NaCl solutions (0.1, 0.2, 0.3, 0.4, 0.5 M). Two major peaks were obtained: one is related to the neutral polysaccharide component separated by water elution and the other is related to the acidic polysaccharide component eluted by 0.1 M NaCl solution. The main component eluted by deionized water was collected, dialyzed overnight, and lyophilized to yield a white flocculent polysaccharide powder. The fraction of CCPP-0 with a higher yield was obtained from DEAE-52 cellulose column chromatography and subsequently subjected to Sephadex G-200 gel filtration to obtain the purified fraction according to molecular weight to obtain homogeneous polysaccharide fractions (Figure 3b). After the in vitro cell pre-experiment, CCPP-0 with better lipid-lowering activity was selected as the main component to explore its lipid-lowering mechanism in subsequent animal experiments.

High-performance gel permeation chromatography (HPGPC) revealed that CCPP-0 is a homogeneous polysaccharide with a molecular weight (Mw) of 47.704 kDa (Table 1), a number-average molecular weight (Mn) of 36.457 kDa, and a polydispersity index (PDI) of 1.309. Its narrow molecular weight distribution (PDI < 1.5) indicates high purity, making it suitable for subsequent animal experiments. CCPP-0 was mainly composed of L-arabinose, D-galactose, and D-glucose (Figure 3c), with a ratio of 1:1.47:9.02, which was measured by high-performance liquid chromatography after precolumn derivatization.

### 3.2. Effects of CCPP on Weight Change and Food Intake in High-Fat Diet-Fed Mice

**Figure 4:**
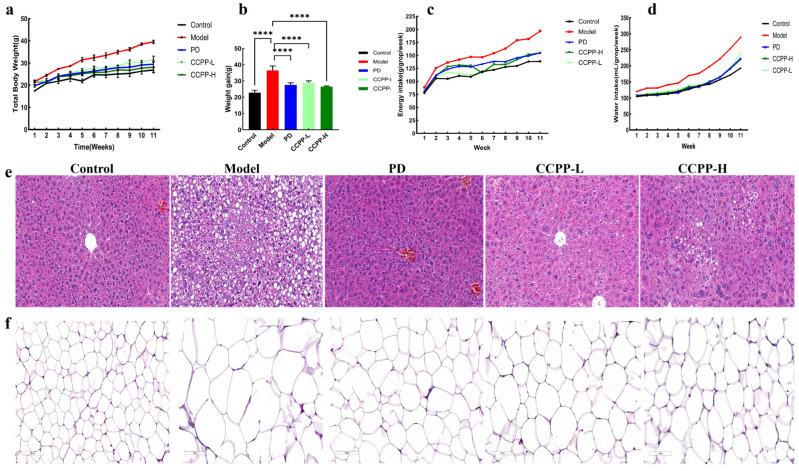
Effects of CCPP on weight, food intake, liver and adipose tissue in high-fat diet-fed mice. (**a**) Total body weight; (**b**) weight gain; (**c**) food intake; (**d**) water intake; *n* = 6. (**e**) H&E staining of liver tissue; (**f**) H&E staining of epididymal white adipose tissue; *n* = 5. **** *p* < 0.0001.

Initial body weight was similar across all five groups (Figure 4a–d), supporting unbiased group assignment. Over three weeks, the Model group showed a marked increase in body weight compared to the Control group. At this time point, the PD, CCPP-L, and CCPP-H groups did not differ significantly from the Model group. Following the 10-week intervention period, however, all three intervention groups (PD, CCPP-L, and CCPP-H) exhibited significantly lower body weight relative to the Model group. After eating high-fat food, the mice began to eat more and drink more water, whereas this trend was significantly reduced following CCPP intervention. The results indicate that CCPP intervention effectively reduced high-fat diet-induced weight gain in mice.

### 3.3. Effects of CCPP on Liver and Adipose Tissue in High-Fat Diet-Fed Mice

Histological examination of liver section (Figure 4e) showed that the structure of the hepatic lobule in the Control group was very clear, with central veins located centrally and hepatic cords arranged radially. Hepatocytes were polygonal with eosinophilic cytoplasm and large round nuclei. The portal areas contained arteries, veins, and bile ducts in normal proportions. Sinusoidal endothelial cells were intact, with scattered Kupffer cells, and no congestion or inflammation was observed. There were no pathological changes such as necrosis, fibrosis, or steatosis. In the Model group, lipid droplet vacuoles (red arrows) were observed in the liver, with large lipid droplets compressing hepatocyte nuclei toward the cell periphery, indicating macrovesicular steatosis. The degree of steatosis varied significantly among the five mice in this group. The liver tissue morphology in the PD group and the CCPP-L group appeared essentially normal, with no significant abnormalities compared to the Control group. In the CCPP-H group, very few to minimal lipid droplet vacuoles were observed under microscopy, with no other notable pathological changes. H&E staining revealed a honeycomb-like structure in the adipose tissue (Figure 4f), composed predominantly of unilocular adipocytes exhibiting polygonal or circular shapes with vacuolated appearances. In the Model group, adipocytes were significantly enlarged with irregular cell sizes and expanded intercellular spaces. The adipocyte size of other subjects is between the Control group and the Model group.

### 3.4. Effects of CCPP on Serum Biochemical Index, Inflammatory Factors, and AST and ALT Enzyme Activities in High-Fat Diet-Fed Mice

#### 3.4.1. Effects of CCPP on Lipid Metabolism in High-Fat Diet-Fed Mice

**Figure 5:**
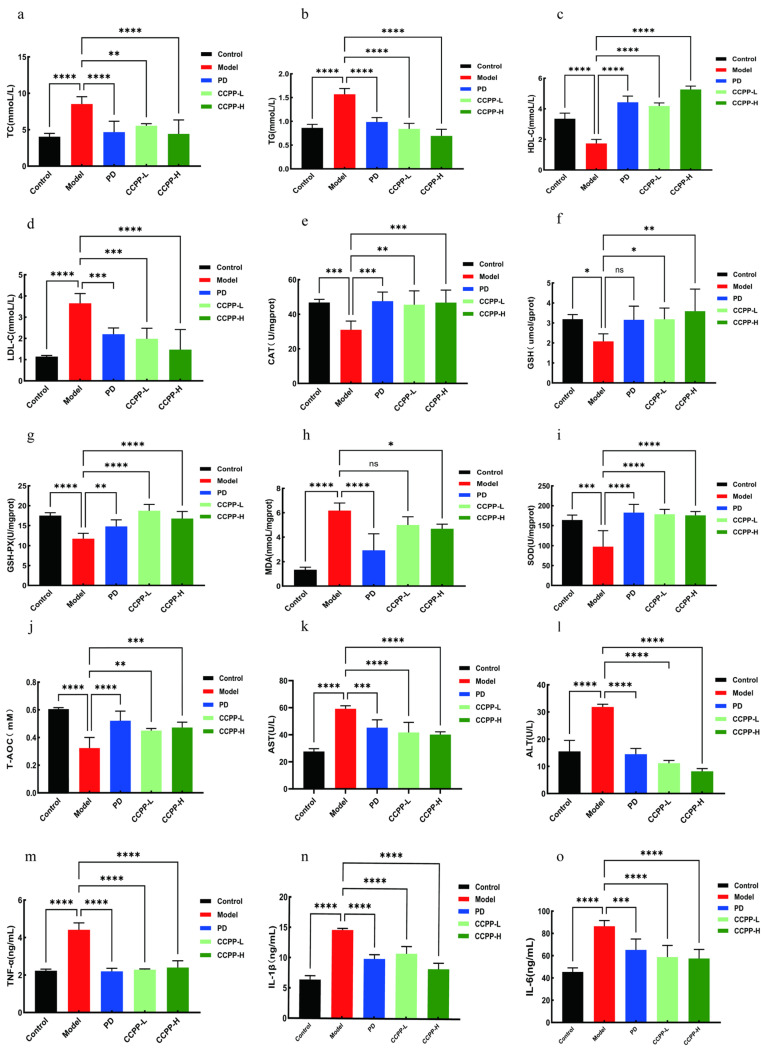
Effect of CCPP on lipid metabolism, oxidative stress, and inflammatory cytokines in high-fat diet-fed mice. (**a**) TC, (**b**) LDL-C, (**c**) TG, (**d**) HDL-C in serum. (**e**) CAT, (**f**) GSH-Px, (**g**) GSH, (**h**) MDA, (**i**) T-AOC, and (**j**) SOD in liver tissue. The effects of CCPP on serum AST and ALT levels in mice (**k**,**l**) and the effect of CCPP on serum inflammatory factors (**m**), IL-1β (**n**), IL-6 (**o**). *n* = 6, “ns” indicates not significant, * *p* < 0.05, ** *p* < 0.01, *** *p* < 0.001, **** *p* < 0.0001.

High triglycerides (TG), elevated low-density lipoprotein cholesterol (LDL-C), and low high-density lipoprotein cholesterol (HDL-C) levels can all increase the risk of atherosclerosis, coronary heart disease, stroke, and other cardiovascular conditions [38,39,40]. Compared with the Control group, mice fed a high-fat diet exhibited elevated serum levels of TC, TG, and LDL-C. After CCPP intervention, the serum levels of TC, TG, and LDL-C were significantly reduced (Figure 5a,b,d). Importantly, compared with the model group, CCPP treatment greatly reduced high-fat diet-induced increases in HDL-C level in the serum of mice (Figure 5c). These findings suggest that CCPP may play a role by maintaining the normal cholesterol transport process, thus helping the body to better regulate blood lipids.

#### 3.4.2. Effects of CCPP on Hepatic Antioxidant Indicators in High-Fat Diet-Fed Mice

A high-fat diet triggers hepatic oxidative stress by increasing the burden of hepatic lipid metabolism, generating excessive reactive oxygen species (ROS), and depleting antioxidant substances. Long-term intake of high-fat foods results in excessive fat accumulation in the liver, resulting in fatty liver disease, which can further progress to non-alcoholic steatohepatitis (NASH). NASH is characterized by hepatic inflammation and oxidative stress, which can damage hepatocytes and lead to liver dysfunction [41,42]. The primary markers of oxidative stress include elevated levels of MDA, accompanied by decreased activities of key antioxidants such as GSH, CAT, and T-AOC [43]. As shown in (Figure 5e–j), our experiment detected changes in hepatic GSH, GSH-Px, SOD, T-AOC, CAT, and MDA. The HFD group exhibited significantly lower concentrations of T-AOC, CAT, GSH, GSH-Px, and SOD compared to the Control group. After CCPP intervention, the HFD + CCPP group showed observably higher concentrations of T-AOC, CAT, GSH, GSH-Px, and SOD, along with lower MDA levels, compared to the HFD group. These findings demonstrate that CCPP enhances the ability to scavenge superoxide anion radicals and significantly alleviates high-fat diet-induced impairment of hepatic antioxidant function.

#### 3.4.3. Effects of CCPP on Serum Inflammatory Factors and AST and ALT Enzyme Activities in High-Fat Diet-Fed Mice

Elevated levels of ALT and AST are the main signs that we use to judge whether liver cells are injured or not. An increase in their levels directly indicates that the integrity of the liver cell membrane has been compromised and that the enzymes have leaked into the bloodstream [44]. As can be seen from Figure 5k,l, the group of small animals that ate high-fat food (HFD) had much higher enzyme indexes representing liver injury, confirming successful model establishment. In contrast, the high-dose CCPP treatment (CCPP-H group) significantly reduced serum ALT and AST levels, restoring them toward the normal range. This indicates that CCPP alleviates diet-induced liver injury. Mechanistically, a high-fat diet promotes hepatic lipid accumulation, steatosis, and inflammation. This lipid overload can subsequently activate inflammatory pathways and trigger the release of pro-inflammatory cytokines [45,46]. The anti-inflammatory potential of CCPP in the context of hyperlipidemia was evaluated by analyzing the serum cytokine profile. As illustrated in (Figure 5m–o). After CCPP intervention, compared with the Model group, the concentrations of TNF-α, IL-1β and IL-6 were significantly decreased. Collectively, the data indicate that CCPP exerts an anti-inflammatory effect within the hepatic tissue of obese mice. The experiment demonstrated that CCPP intervention significantly alleviated the inflammatory response in mice with hyperlipidemia, indicating its potential role in the regulation of inflammation.

### 3.5. CCPP Restore the Production of SCFAs in the Cecum in High-Fat Diet-Fed Mice

**Figure 6:**
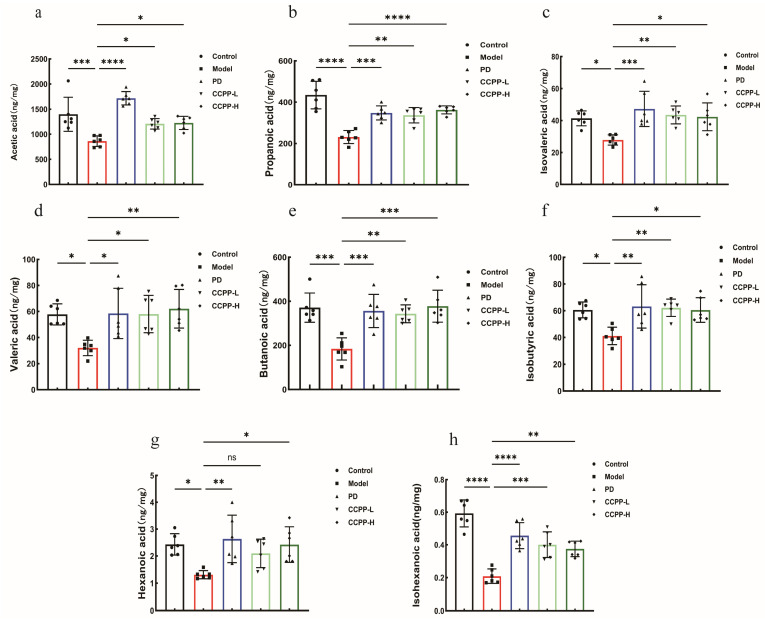
Effects of CCPP intervention on short-chain fatty acids (SCFAs) content in the colon of HFD-fed mice: acetate (**a**); propionate (**b**); isovalerate (**c**); valerate (**d**); butyrate (**e**); isobutyrate (**f**); caproate (**g**); isocaproate (**h**). (*n* = 6), “ns” indicates not significant, * *p* < 0.05, ** *p* < 0.01, *** *p* < 0.001, **** *p* < 0.0001.

Intestinal microorganisms will produce short-chain fatty acids as by-products when they decompose dietary fiber, playing important roles in host physiological functions. These fatty acids primarily include acetate, propionate, isobutyrate, butyrate, isovalerate, and valerate [47]. SCFAs are associated with metabolic syndrome and are involved in the regulation of energy metabolism [48]. They contribute to maintaining intestinal barrier integrity [49], modulating gut immune function [50], and play significant roles in cardiovascular diseases [38], including hypertension [51], ischemia–reperfusion injury, myocardial infarction, atherosclerosis, and heart failure [52]. Analysis of SCFAs levels in high-fat diet (HFD)-fed mice revealed that CCPP intervention significantly ameliorated gut microbial metabolic disorders. Compared to the HFD group, the CCPP group exhibited significantly increased levels of acetate and butyrate. Furthermore, propionate, caproate, and valerate levels were also markedly elevated after CCPP intervention (Figure 6). Notably, the reduced levels of isobutyrate, isocaproate, and isovalerate in HFD mice were significantly restored following CCPP intervention. These findings suggest that CCPP may alleviate high-fat diet-induced obesity and related metabolic disorders by remodeling the gut microbiota metabolic profile, particularly through promoting the production of SCFAs.

### 3.6. CCPP Supplementation Modulated Gut Microbiota in High-Fat Diet-Fed Mice

**Figure 7:**
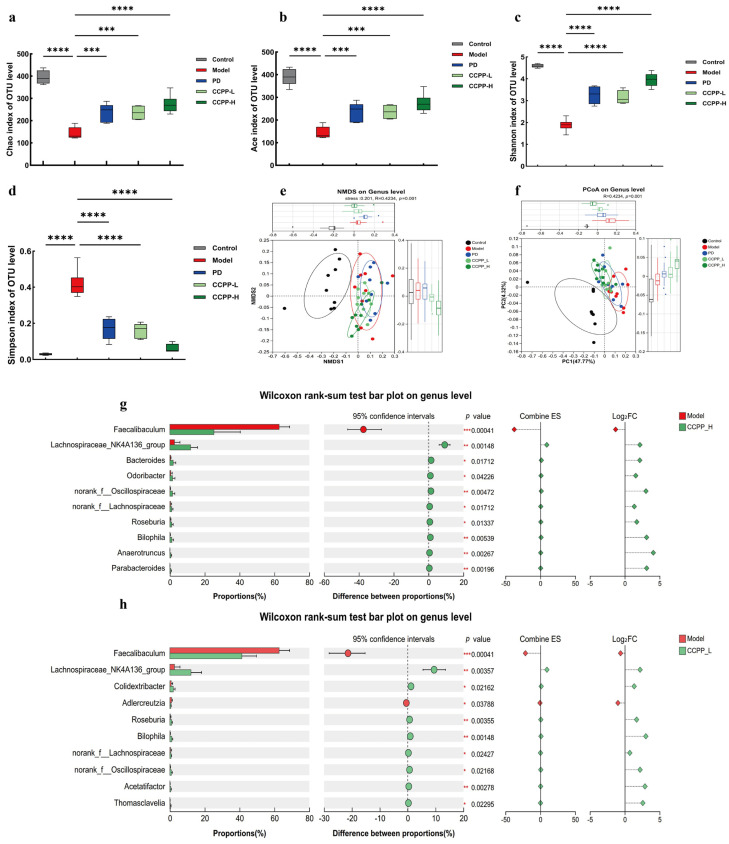
Effects of CCPP supplementation on Alpha and Beta diversity of fecal microbiota in each group of mice. (**a**) Chao index, (**b**) ACE index, (**c**) Shannon, (**d**) Simpson index, (**e**) NMDS, and (**f**) PCoA, (**g**) The differential species between the CCPP-H group and the Model group. (**h**) The differential species between the CCPP-L group and the Model group. * *p* < 0.05, ** *p* < 0.01, *** *p* < 0.001, **** *p* < 0.0001.

The gut microbiota, often referred to as a “special organ” for host health, plays a critical role in functions such as substance metabolism and immune regulation [34]. Polysaccharides, as a cornerstone of dietary fiber, have the ability to shape both the makeup and activity of gut microbes through the process of bacterial fermentation, as they are resistant to enzymatic hydrolysis in the host’s digestive tract [53]. Relevant research findings indicate that patients with hyperlipidemia often exhibit gut microbiota dysbiosis [54,55,56]. The alpha diversity index highlights differences in microbial communities across groups. With a high average coverage index of 0.999 across all five groups, the sequencing data reliably represents the in vivo composition of the gut microbiota. AS shown in (Figure 7a–d), compared with the Control group, the ACE, Chao, Shannon, and index values in the Model group were significantly lower, while the Simpson index was significantly higher; compared with the Model group, the ACE, Chao, and Shannon index values in the CCPP group were significantly increased, while the Simpson index was significantly decreased, and all metrics aligned with the Control group’s data. The findings reveal that a high-fat diet takes a toll on the gut microbiome of mice, reducing both its richness and diversity. On the flip side, CCPP supplementation appears to turn the tide, effectively boosting microbial diversity and abundance in mice maintained on such a diet. Multivariate statistical analyses, including PCoA (Figure 7f) and NMDS (Figure 7e), elucidated that HFD-fed mice significantly altered microbial communities, and CCPP supplementation could reverse the altered gut microbiota to a certain extent. We employed the Wilcoxon rank-sum test to compare species abundance across the two groups [57]. The results showed that the Model group mice exhibited a higher prevalence of harmful bacteria, while the growth of beneficial bacteria was inhibited. It has been demonstrated that sugar components in a high-fat diet (HFD), such as sucrose and maltose, are key factors responsible for the reduction of intestinal commensal T helper 17 cells, with sugar displacing segmented filamentous bacteria (SFB) through increased abundance of *Faecalibaculum rodentium*. This finding is consistent with similar observations made in the HFD model group, as illustrated in (Figure 7g,h). In contrast, CCPP intervention significantly increased the abundance of beneficial, short-chain fatty acid-producing bacteria, particularly the *Lachnospiraceae_NK4A136_group*, which are crucial for gut health and metabolic regulation. This modulation of the gut microbiota composition represents a key pathway through which CCPP exerts its therapeutic effects.

**Figure 8:**
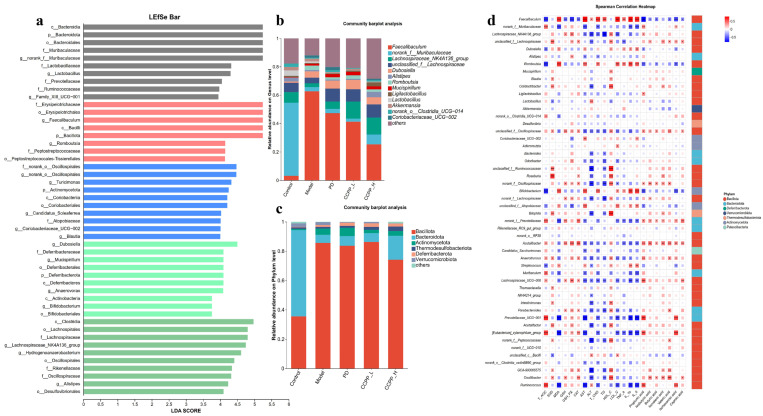
Effects of CCPP on species composition of gut microbiota and Spearman correlation analysis. (**a**) LEfSe analysis, (**b**) relative abundance of fecal microbiota at the phylum level in mice fed an HFD, (**c**) relative abundance of fecal microbiota at the genus level in mice fed an HFD, (**d**) heatmap of Spearman correlation of indicators among the relative abundance of bacterial genera in the gut microbiota and lipid metabolism or inflammatory indicators. Only taxa meeting an LDA score threshold of >2 are shown. * *p* < 0.05, ** *p* < 0.01, *** *p* < 0.001.

As shown in (Figure 8a), LEfSe analysis revealed that the intestinal microbiota in the Control group was characterized by a significant enrichment of beneficial bacteria, with Bacteroidia being particularly prominent. In contrast, the Model group exhibited a relative enrichment of harmful bacteria, represented by *Erysipelotrichaceae*. The dominant microbial community in the PD intervention group primarily consisted of *Oscillospiraceae*. In the CCPP-L group, beneficial bacteria represented by *Dubosiella* emerged as the predominant enriched taxa, while in the CCPP-H group, the *Lachnospiraceae_NK4A136_group* was notably abundant. Compared to the Control group, the relative abundance of the phylum *Bacillota* was significantly increased in the Model group, while that of *Bacteroidota* showed a significant decrease. CCPP supplementation intervene significantly enriched beneficial gut microbiota in high-fat diet-fed mice, thereby ameliorating the progression of obesity and lipid metabolism. This study further investigated how CCPP intervention alters gut microbiota composition in mice fed with a high-fat diet. Specifically, the relative abundances of microbial taxa were analyzed and compared at both the phylum and genus levels among the five experimental groups. The taxonomic composition of the gut microbiota at the phylum level for all five groups is shown in (Figure 8b). The gut microbiota of mice was primarily composed of microorganisms from the phyla *Bacillota*, *Bacteroidota*, *Actinobacteriota*, *Thermodesulfobacteriota*, and *Verrucomicrobiota*. Compared with the Model group, the relative abundance of *Bacteroidota*, *Verrucomicrobiota*, and *Actinomycetota* in all CCPP intervention groups showed a dose-dependent increase, whereas that of *Bacillota* and exhibited a dose-dependent decrease. In addition, compared with both the Model and Control groups, the relative abundance of *Bacillota* was significantly reduced in the low- and high-dose CCPP intervention groups. Analysis of (Figure 8c) revealed distinct differences in genus-level microbial abundance among fecal samples from the different mouse groups. The Control group was predominantly characterized by *Muribaculaceae*, *Lachnospiraceae_NK4A136_group*, and *Lachnospiraceae*. On the contrary, the Model group showed higher relative abundances of *Mucispirillum*, *Faecalibaculum*, and *Dubosiella*, along with lower proportions of *Lactobacillus* and *Bacteroides*. Compared to the Control group, the Model group showed reduced abundances of both *Lactobacillus* and *Bifidobacterium*, while the abundance of *Faecalibaculum* was significantly increased. As illustrated in Figure 8c, the relative abundances of *norank_f__Muribaculaceae*, *Lachnospiraceae_NK4A136_group* and *unclassified_f__Lachnospiraceae* were significantly higher in the CCPP intervention groups than in the Model group. In contrast, the relative abundance of *Faecalibaculum* and *Romboutsia* was significantly reduced, exhibiting a dose-dependent decrease with CCPP intervention. Spearman correlation analysis was performed to evaluate the relationships between gut microbiota and various host parameters, including lipid profiles, inflammatory cytokines, antioxidant indices, and SCFAs. The result shown in (Figure 8d) in the *Lachnospiraceae_NK4A136_group* displayed negative correlations with TG, LDL-C, TNF-α, IL-1β, and IL-6 levels, while demonstrating positive correlations with HDL-C and the concentrations of propionate, isovalerate, valerate, butyrate, and isobutyrate. *Roseburia* was positively correlated with HDL-C and the levels of caproate, isocaproate, propionate, isovalerate, valerate, butyrate, and isobutyrate. These results indicated that a high-fat diet reduces the relative abundances of *Lactobacillus* and *Bifidobacterium* in the gut microbiota of mice. CCPP intervention, however, modulated the relative abundances of beneficial bacteria such as *Lachnospiraceae*, *Bacteroides*, and *Lactobacillus* toward normal levels, while reducing the relative abundance of *Faecalibaculum*, thereby contributing to the restoration of healthy gut microbiota.

## 4. Discussion

As global living standards have risen, obesity rates have climbed significantly, contributing to a growing burden of related diseases worldwide [58]. Obesity is a complex chronic metabolic disorder that can predispose individuals to other metabolic diseases, such as type 2 diabetes, non-alcoholic fatty liver disease, and cardiovascular diseases [59]. Traditional anti-obesity pharmacotherapies, while effective, are often associated with adverse side effects, underscoring the need for safer, more sustainable interventions. This has led to an increased focus on natural substance sources and their favorable safety profiles [60]. Lipid metabolism plays a crucial role in maintaining fundamental life activities, and developing natural therapeutics for lipid-related disorders has become a current trend [61]. Lipid metabolism can be modulated by natural plant polysaccharides through gut microbiota-mediated pathways, which are associated with enhanced barrier function, reduced inflammation, and inhibition of pathogenic bacteria, decreasing Trimethylamine N-Oxide production, and modulating gut microbial metabolites like SCFAs and bile acids/bile acid salts for improved human health [62]. While considerable advances have been made in understanding how polysaccharides modulate lipid metabolism via gut microbiota, key challenges remain unaddressed.

In this study, a mouse model was established using a high-fat diet (HFD). After 10 weeks of HFD feeding, compared with the control group, the HFD group exhibited hyperplasia and hypertrophy of white adipose tissue, increased lipid accumulation in the liver, and noticeable hepatocyte damage. Additionally, serum antioxidant enzyme activity was significantly decreased, while the levels of pro-inflammatory cytokines (TNF-α, IL-1β, and IL-6) were markedly elevated. However, after CCPP intervention in high-fat diet-induced mice, the serum levels of TC, TG, and LDL-C were significantly reduced, while the HDL-C level was markedly increased. Likewise, CCPP intervention demonstrated a comparable dose-dependent trend, further supporting the notion that the biological efficacy of such polysaccharides is intrinsically linked to dosage. Consistent with our findings, Zhang et al. [63] reported that Morchella esculenta polysaccharide (MEP-1) significantly ameliorated lipid metabolic disorders in high-fat diet (HFD)-fed mice. MEP-1 administration dose-dependently reduced serum levels of total cholesterol (TC), triglycerides (TG), low-density lipoprotein cholesterol (LDL-C), and the pro-inflammatory cytokines TNF-α, IL-1β, and IL-6, with the most pronounced effects observed at the high dose (400 mg/kg/day). This clearly demonstrates a dose–response relationship in lipid regulation by polysaccharides. Moreover, Ilex cornuta tea polysaccharide (GTP40) has also been shown to exert dose-dependent effects against HFD-induced hyperlipidemia by suppressing abnormal body weight gain and improving dyslipidemia. These independent observations collectively underscore the consistent, dose-dependent therapeutic potential of polysaccharides in modulating metabolic disorders [54]. The alleviation of dyslipidemia in hyperlipidemic mice following CCPP intervention observed here is supportive of the prior findings of Li et al. [64]. ALT and AST are two important enzymes that are present in hepatocytes. When liver cells are damaged, these enzymes are released into the bloodstream, leading to elevated serum enzyme activity [65]. Therefore, the measurement of serum ALT and AST levels is commonly used in clinical practice to assess liver health [66]. The experimental results revealed that serum levels of ALT and AST were significantly elevated in the Model group, whereas CCPP intervention markedly reduced the activities of these enzymes. This indicates that CCPP possesses free radical-scavenging and antioxidant effects, which is largely consistent with the findings reported by Zhang et al. [67]. In our analysis of inflammatory cytokine levels, we found that CCPP intervention reduced the concentrations of pro-inflammatory factors (TNF-α, IL-1β, and IL-6) and alleviated inflammatory responses associated with obesity development. H&E staining of liver tissue and epididymal white adipose tissue revealed that a high-fat diet induced significant hepatic lipid accumulation and adipocyte vacuolization, while CCPP intervention markedly ameliorated these pathological changes, with no significant differences observed compared to the Control group.

Relevant studies indicate that polysaccharides can promote the production of short-chain fatty acids, thereby contributing to the repair of intestinal barrier damage caused by a high-fat diet [68]. Li et al. [69] demonstrated that broccoli ameliorates lipid metabolism and modulates gut microbiota in type 2 diabetic mice. Specifically, broccoli-derived polysaccharides enhanced the production of SCFAs, thereby exerting beneficial effects on intestinal health. Consistent with these findings, our study showed that CCPP intervention significantly increased the levels of propionate, caproate, and valerate in high-fat diet-fed mice. Notably, the reduced levels of isobutyrate, isocaproate, and isovalerate in HFD-fed mice were also significantly reversed following CCPP treatment. These findings indicate that CCPP alleviates high-fat diet-induced obesity and related metabolic disorders by remodeling the gut microbiota metabolic profile, particularly via promoting beneficial short-chain fatty acid (SCFA) production (Figure 7). Consequently, the restoration of SCFA levels represents a key mechanistic pathway through which CCPP exerts its therapeutic effects. Current research indicates that hyperlipidemic patients often exhibit gut microbiota dysbiosis, and a similar phenomenon has been observed in mice with reduced gut microbial abundance [70,71,72]. Our findings demonstrated a consistent trend that the Model group exhibited a significant reduction in microbial diversity compared to the Control group. Following CCPP intervention, the Simpson index decreased, while the Shannon, Chao, and ACE indices increased significantly. A growing body of evidence demonstrates that polysaccharide supplementation can induce substantial shifts in the gut microbial community, often correlating with improved metabolic parameters. For instance, polysaccharides derived from *Plantago asiatica* L. seeds have been shown to reduce serum triglycerides, cholesterol, and the atherogenic index in mice. Concurrently, these polysaccharides lowered hepatic total lipids and cholesterol levels and modulated the colonic microbiota [73]. Similarly, polysaccharides from Nostoc sphaeroides Kütz and its powder formulation were found to enrich the core bacterial communities in C57BL/6 mice. 16S rRNA gene sequencing revealed that both treatments altered the gut microbiota composition by increasing the relative abundances of the phyla Firmicutes and Bacteroidetes while reducing the abundance of Proteobacteria [74]. Given that a high-fat diet (HFD) is a common inducer of gut dysbiosis, the potential of various polysaccharides to counteract this effect is of considerable interest. Cordyceps militaris polysaccharides, for example, alleviated HFD/high-sucrose diet-induced obesity and hyperlipidemia in mice and corrected the associated imbalance in the gut microbiota [75]. In HFD-induced type 2 diabetic (T2DM) mice, Morchella esculenta polysaccharide (MEP) effectively ameliorated hyperglycemia and hyperlipidemia. This was accompanied by a beneficial modulation of the gut microbiota, which notably increased the abundances of beneficial bacteria such as *Lactobacillus* and *Bifidobacterium* [76,77]. This aligns with our findings, as CCPP intervention was able to modulate the relative abundance of beneficial bacteria such as *Lachnospiraceae*, *Bacteroides*, and *Lactobacillus* in the gut microbiota of high-fat diet-fed mice, thereby ameliorating hyperlipidemia in the mice. Polysaccharides isolated from WuGuChong have also been shown to exert anti-obesity effects in HFD-fed mice, which are mediated by the regulation of gut microbiota composition [78]. Notably, polysaccharides extracted from *Hericium erinaceus*—both prior to and following fermentation—have displayed protective effects on the intestinal barrier while eliciting substantial alterations in gut microbiota structure [79]. Of additional relevance, *Black Lycium barbarum polysaccharide (BLBP)* has been demonstrated to alleviate lipopolysaccharide-induced intestinal injury in mice, an effect associated with its capacity to modulate the gut microbial community [80]. CCPP administration reduced the abundance of *Thermodesulfobacteriota* and *Verrucomicrobiota*, thereby improving the composition of the gut microbiota, which is largely consistent with previous research findings [81]. To sum up, the structural and compositional changes in the intestinal microbiota caused by a high-fat diet, as well as the alterations in species richness and diversity, could be reversed to a certain extent by CCPP.

The significant improvements in the body weight, lipid profile, and systemic inflammation observed in our HFD-fed mice following CCPP intervention highlight its therapeutic potential. While prescription anti-obesity drugs are effective, they are frequently associated with adverse effects ranging from mild gastrointestinal discomfort to more severe risks, which can compromise treatment sustainability. For instance, the reliance on single-target mechanisms may also contribute to limited efficacy or compensatory adaptations in complex metabolic networks. In contrast, our findings, consistent with other studies on plant polysaccharides [82,83], suggest that CCPP exerts its benefits through a multi-target, system-level approach, primarily by remodeling the gut microbiota and enhancing SCFAs production. This prebiotic-like, holistic mode of action likely underlies its observed efficacy without apparent adverse effects in our model, aligning with the general safety record of dietary polysaccharides. Thus, CCPP represents a promising complementary strategy that may circumvent some limitations of current drugs, offering a favorable risk–benefit ratio for long-term management of obesity and metabolic syndrome.

This study primarily focused on elucidating the upstream mechanism—specifically the “microbiota–SCFAs axis”—by which the intervention modulates lipid metabolism disorders via the gut microbiota and its metabolites. This provides a solid mechanistic foundation for understanding the related metabolic regulation. However, several limitations should be noted. Most notably, oral glucose tolerance tests (OGTTs), insulin tolerance tests (ITTs), and systemic insulin measurements were not performed in all experimental subjects. Consequently, whether and how the core mechanism identified here directly affects systemic glucose metabolic homeostasis—particularly insulin resistance and glucose stability—remains to be experimentally verified. This represents an important limitation of the present work. Future studies will address the following two interrelated aspects. First, dedicated experiments will be designed to systematically evaluate the effects of the intervention on glucose tolerance and insulin sensitivity. This will serve as an important independent direction to investigate the role of this mechanism in glucose metabolism regulation. Second, to more comprehensively map the metabolic profile, systematic lipidomics analyses will be conducted. These analyses aim to reveal detailed changes in specific host lipid species, such as long-chain fatty acids, under the regulation of this mechanism. Together, these approaches will offer a more thorough understanding of the metabolic pathway involved.

## 5. Conclusions

This study extracted coffee cherry peel polysaccharides (CCPPs) via ultrasound-assisted hot water extraction and alcohol precipitation, then evaluated their effects on high-fat diet (HFD)-induced obese mice. The results showed that CCPP significantly reduced body weight, food intake, hepatic fat accumulation, and white adipose tissue size while lowering serum TC, TG, and LDL-C to exert lipid-lowering effects. It enhanced hepatic antioxidant enzyme (SOD, CAT, GSH-Px, T-AOC) activities, increased GSH content, decreased MDA levels, and reduced the number of pro-inflammatory cytokines (TNF-α, IL-1β, IL-6) present, alleviating oxidative stress and inflammation. Additionally, CCPP regulated gut microbiota composition and promoted beneficial short-chain fatty acid (SCFA) production. Collectively, CCPP exhibits prebiotic potential in preventing diet-induced obesity and mitigating HFD-related metabolic disorders, supporting its application in functional food development and dietary management of lipid metabolism disorders.

## Figures and Tables

**Table 1:** Molecular weight of polysaccharides from fresh coffee cherry peel.

Sample Name	Mw (kDa)	Mn (kDa)	Mw/Mn (kDa)
CCPP-0	47.704	36.457	1.309

## Data Availability

The original contributions presented in this study are included in the article. Further inquiries can be directed to the corresponding authors.
